# Necroptosis: is there a role for mitochondria?

**DOI:** 10.3389/fphys.2014.00323

**Published:** 2014-08-26

**Authors:** Kurt D. Marshall, Christopher P. Baines

**Affiliations:** ^1^Department of Biomedical Sciences, University of Missouri-ColumbiaColumbia, MO, USA; ^2^Dalton Cardiovascular Research Center, University of Missouri-ColumbiaColumbia, MO, USA; ^3^Department of Medical Pharmacology and Physiology, University of Missouri-ColumbiaColumbia, MO, USA

**Keywords:** necrosis, necroptosis, mitochondria, reactive oxygen species, TNFα

## Abstract

Once thought to be a random process of cell death, necrosis can proceed via a defined molecular mechanism and is integral to physiological and pathological states. In particular a form of necrosis called necroptosis has been the subject of intense investigation. Necroptosis is initiated by tumor necrosis factor-α (TNFα), which leads to the activation of the kinase receptor-interacting protein 1 (RIP1). RIP1 then binds with and activates RIP3 to form the necrosome. RIP3 in turn interacts with and activates the pseudokinase mixed lineage kinase domain-like (MLKL). This complex has then been proposed to induce necrotic death via the induction of mitochondrial dysfunction, with a variety of mechanisms being put forth including: production of mitochondrial reactive oxygen species (ROS), activation of the mitochondrial phosphatase PGAM5, or induction of mitochondrial permeability transition (MPT). However, recent evidence suggests that none of these are involved in necroptosis, and that mitochondria may in fact be dispensable for this process. Therefore, the purpose of this perspective is to discuss the current understanding of necroptosis, and more specifically, what role if any do mitochondria play in this mechanism of cell death.

## Introduction

Cells die for a variety of reasons and under myriad circumstances. Cells are thought to undergo three major forms of regulated cell death: apoptosis, autophagy, and necrosis. In contrast to apoptosis and autophagy, necrosis was once thought of as a random uncontrolled form of cell death. However, it has been established that necrosis can proceed via a controlled and varied series of events. The most studied and defined molecular necrotic pathway is traditionally mediated by signaling by the tumor necrosis factor-α (TNFα) receptor (TNFR) through RIPs 1 and 3 and subsequently the pseudokinase MLKL and is termed necroptosis (Christofferson et al., 2014; Galluzzi et al., 2014; Vanden Berghe et al., 2014).

From its earliest description, execution of necroptosis has been thought to involve ROS and mitochondria (Van Herreweghe et al., 2010). This assumption has been perpetuated by countless studies in the nearly 10 years since the first use of the term necroptosis. Recent evidence casts doubt on the idea that signaling via the necrosome requires ROS and mitochondria. In fact, cells are able to undergo necroptosis in the near absence of mitochondria and ROS is dispensable for this event (Tait et al., 2013).

While the proximal molecular componentry of the necroptotic pathway is known, its downstream signaling, until recently, has been poorly understood. Although mitochondria in a variety of guises have been proposed to be mediators of necroptosis, recent data has suggested that they may not in fact be necessary for the execution of the necroptotic program. This perspective will therefore summarize the current understanding of necroptosis signaling and discuss whether mitochondria have any role to play in this form of programmed necrosis.

## Programmed necrosis and the necrosome

The pro-inflammatory cytokine TNFα plays an important role in inducing cell death (Van Herreweghe et al., 2010; Vanden Berghe et al., 2014). In response to TNFα stimulation, TNFR recruits proteins to the plasma membrane (Van Herreweghe et al., 2010; Christofferson et al., 2014; Vanden Berghe et al., 2014). When TNFR trimerizes in response to ligand binding, TNFR1-associated death domain protein (TRADD) is recruited to the cytoplasmic domain of this receptor (Hsu et al., 1995). The TNFR/TRADD complex then recruits RIP1 and TNFR-associated factor 2/5 (TRAF2/5) to the plasma membrane (Hsu et al., 1996a,b; Tada et al., 2001; Ermolaeva et al., 2008). Next, cellular inhibitor of apoptosis proteins (cIAP1/cIAP2) are recruited to the TNFR which are involved in inhibition of RIP1 and RIP3 signaling (McComb et al., 2012). Together with the TNFR, this assemblage of proteins is known as complex I, and is responsible for signaling a variety of cell behaviors including proliferation, survival and NF-κ B signaling (Christofferson et al., 2014). Complex I can transition from a membrane associated protein assembly to the cytosol. This cytosolic collection of proteins is known as complex IIa and in place of TNFR from complex I contains caspase 8 and adaptor protein FADD (Christofferson et al., 2014; Vanden Berghe et al., 2014). Formation of complex IIa can yield two cell fates, apoptosis or necrosis, and when caspase 8 is inhibited, necrosis prevails.

Through the interaction of RIP1 and RIP3 necroptotic signaling is induced (Cho et al., 2009; He et al., 2009; Zhang et al., 2009). RIP1 and RIP3 comprise the necrosome, on which this perspective will be focused. Post-translational modifications of RIP1 and RIP3 are crucial steps for the initiation of necrosome formation and signaling (Cho et al., 2009; He et al., 2009; Zhang et al., 2009). Initially, cylindromatosis (CYLD) a deubiquinase has been shown to deubiquinate RIP1 after complex I dissociates from the TNFR and facilitate necrosome signaling (Moquin et al., 2013). This deubiquitination activates RIP1 so that it can bind to and phosphorylate RIP3. This activates RIP3, whose kinase activity is required for necroptotic signaling (Cho et al., 2009; He et al., 2009; Newton et al., 2014). Recent experiments have begun to elucidate signaling events in programmed necrosis downstream of the necrosome. Specifically, RIP3 dependent phosphorylation of the pseudokinase mixed lineage kinase domain-like protein (MLKL) has been shown to be essential for RIP1/RIP3-dependent necroptosis (Sun et al., 2012; Zhao et al., 2012; Murphy et al., 2013). However, at this point the sequence of events downstream of RIP1/RIP3/MLKL that ultimately lead to cell rupture becomes muddled. Contradictory experimental results point toward two different mechanisms of necroptosis, either dependent on the mitochondria through a variety of mechanisms, or independent of these events. The remainder of this perspective will focus on the evidence supporting these two divergent hypotheses.

## The role of mitochondria in necroptosis: evidence for

Necrosomal activation is known to induce programmed necrosis, however new results call into question the mechanism by which this process progresses. Classically, necrosomal signaling was thought to involve ROS generation from the mitochondria in the execution of cell death. An association between necrosome signaling and ROS generation is illustrated in many studies. The complex II and necrosome proteins RIP, TRAFF, and FADD have been shown to be critical for the accumulation of ROS in TNFα signaling (Lin et al., 2004). In this exploration of necroptotic signaling, mouse embryonic fibroblasts (MEFs) (WT, RIP1^−/−^, TRAFF^−/−^ and FADD^−/−^) were exposed to TNFα, and ROS was measured by dichlorofluorescin diacetate (DCFDA). From their data, it was proposed that necroptotic signaling proceeds through the TNFα-induced accumulation of ROS. The necessity of ROS for this process was confirmed via the use of the antioxidant butylated hydroxyanisole, which blocked ROS accumulation and the comsensurate cell death. TNFα mediated ROS generation has been shown to be dependent on RIP1 and mitochondrially derived in L929 cells (Vanlangenakker et al., 2011). This was determined by inhibiting cytoplasmic ROS generation by siRNA mediated knockdown of NADPH oxidase components, which did not affect TNFα mediated cell death. However, with suppression of complex I of the respiratory chain, TNFα mediated cell death was attenuated. In support of this finding, mitochondrial, but not cytosolic ROS is critical in mediating TNFα induced cell death in L929 and RAW 264.7 cells (Ardestani et al., 2013). These data confirmed the results of Vanlangenakker in L929 cells and extended this finding to a different cell type, a mouse monocyte line. Studies have indicated that complex I of the electron transport chain is responsible for the ROS production seen during TNFα-induced necroptosis (Schulze-Osthoff et al., 1992; Goossens et al., 1999). In both of these studies, ROS generation was measured in L929 cells. This coupled with the fact that RIP1, RIP3, and/or MLKL have all been reported to translocate to the mitochondrial fraction upon stimulation in a variety of cell types (Temkin et al., 2006; Zhang et al., 2009; Davis et al., 2010; Wang et al., 2012), indicated that ROS production could indeed be a key step in the execution of the necroptotic process. In addition to showing mitochondrial translocation of RIP3 to the mitochondria in MEFs, Davis et al. were able to inhibit necrotic cell death in endothelial cells with the mitochondrial antioxidant MnSOD (Davis et al., 2010). From the current data, it is clear that certain cell types, such as L929 cells, are heavily represented in the study of necroptosis. However, it is important to note that mitochondrial ROS is implicated in TNFα induced necrosis by several groups using other cell types (MEFs, endothelial cells and RAW 264.7 cells).

Another potential mitochondrial mediator of necroptosis and one that may provide a link to ROS production is the mitochondrial permeability transition (MPT) pore. The MPT pore is a large, non-specific channel that spans the inner mitochondrial membrane. Opening of the MPT pore leads to a loss of the mitochondrial transmembrane potential and failure of oxidative phosphorylation, production of ROS, and ultimately swelling and rupture of the organelle (Baines, 2010). Genetic experiments where a critical regulator of the MPT pore, cyclophilin-D (CypD), was knocked out revealed a role for the MPT pore primarily in necrotic cell death as opposed to apoptosis (Baines et al., 2005; Nakagawa et al., 2005; Schinzel et al., 2005). Whether the MPT pore couples to necroptotic signaling has been the subject of considerable study. TNFα-induced necroptosis was found to be partially attenuated by the loss of CypD in mouse embryonic fibroblasts (He et al., 2009). Similarly, TNFα-induced zebrafish macrophage ROS production and necrosis was blocked by the CypD inhibitor alisporivir (Roca and Ramakrishnan, 2013). In the myocardium, protection against ischemia/reperfusion by the RIP1 inhibitor necrostatin was not additive to that conferred by CypD ablation, also suggesting that the 2 components were part of the same genetic pathway (Lim et al., 2007).

There is also evidence the other mitochondrial functions/components may play a role in TNFα-induced necroptosis. For example, Zhang et al. (2009) found that RIP3 could translocate to the mitochondria where it interacted with the mitochondrial protein glutamate dehydrogenase 1 (GLUD1). Consistent with this they demonstrated that knockdown of GLUD1 could at least partially block TNFα-induced ROS production as measured by DCFDA staining and necrosis in NIH 3T3 cells. Bcl2 family proteins may also play a role in necroptosis. Specifically, Hitomi et al. (2008) identified the Bcl2 protein Bmf in a proteomic screen for potential mediators of TNFα-induced necroptosis, and demonstrated that at least in L929 cells depletion of Bmf could attenuate the necrotic response to TNFα. That being said, how exactly Bmf is functioning at the mitochondria and whether this is a conserved mechanism in other cell types has yet to be tested.

Finally, the interaction of RIP3 with MLKL has been reported to induce translocation of the RIP1/RIP3/MLKL complex to the mitochondrial membrane as TNFα induced necroptosis results in enriched levels of RIP1/RIP3/MLKL in the mitochondrial associated membrane fraction of cells, i.e., the contact sites between the outer mitochondrial membrane and the ER membrane (Chen et al., 2013). Upon translocation to the mitochondria, the necrosome interacts with and activates the mitochondrial phosphatase PGAM5 (Wang et al., 2012). Signaling of the necrosome through PGAM5 was then shown to result in mitochondrial fragmentation in a Dynamin-related protein 1 (Drp1) manner (Wang et al., 2012). These data were obtained in HeLa cells, and indicated a direct interaction between RIP1/RIP3 and PGAM5 as determined by immunoprecipitation. siRNA mediated knockdown of Drp1 and inhibition of Drp1 with mdivi-1 were able to inhibit TNF mediated necroptosis in both HeLa and HT-29 cells. In further support of this, Drp1 depletion decreases death in NRK-52E (rat renal tubule epithelial cells) treated with TNFα (Zhang et al., 2013).

## The role of mitochondria in necroptosis: evidence against

The studies described above all suggested that in one form or another, mitochondria played a pivotal role in the execution of the necroptotic program. However, several recent studies have questioned the role of each of the mitochondrial facets in necroptosis and suggest that mitochondria maybe dispensable for this process. For example, many early studies proposing a role for mitochondrial ROS merely show a circumstantial relationship between ROS, mitochondria and cell death. In their description of RIP3 as a component of the necrosome, He et al. called into question the necessity for ROS in necrosome signaling (He et al., 2009). Their results indicate that the requirement of ROS in necrosome mediated cell death may be cell type specific, where ROS scavenging protects L929 but not HT-29 cells from TNFα-induced necrotic cell death. Macrophage and Jurkat cell necrosis appears to be ROS-independent (Moquin and Chan, 2010), indicating that ROS are not absolutely required for necroptosis. In confirmation of this, the human monocyte derived THP-1 cell line shows an increase in ROS in response to TNF treatment, but treatment with a variety of antioxidants (ascorbic acid, glutathione, and *N*-acetly-cystein) does not attenuate death (Temkin et al., 2006).

Whether the MPT pore plays a role in necroptosis has also been questioned. Specifically, the embryonic lethality caused by caspase-8 deletion is due to RIP3-dependent necroptosis (Kaiser et al., 2011), and cannot be rescued by CypD ablation (Tait et al., 2013). Similarly, necroptosis in caspase-8 deficient macrophages could be blocked by depletion of RIP1 and RIP3 but not by depletion of CypD (Ch'en et al., 2011). Finally, a recent paper examining ischemia/reperfusion injury in the kidney found that ablation of RIP3 and CypD was protective but that double knockout mice exhibited even great protection (Linkermann et al., 2013), suggesting that the necrosome and the MPT pore are distinct pathways. This is opposite to that seen in the heart and raises the question of whether there are tissue-specific differences in the functional coupling of the MPT pore to RIP1-dependent signaling. Moreover, although CypD is a major regulator of the pore, it is not the pore itself and it maybe that necroptotic signaling can bypass CypD and directly activate the pore; in this scenario loss of CypD would not be expected to offer protection against caspase-8 deletion. Both of these issues need to be examined further.

The role of the mitochondrial PGAM5-Drp1 axis has also been questioned by several recent studies. Silencing of PGAM5 was found to have no effect on necroptosis induced by TNFα or RIP3 dimerization in a variety of cell lines (Murphy et al., 2013; Tait et al., 2013; Remijsen et al., 2014). Murphy et al. found that knocking down PGAM5 with shRNA in MEFs did not attenuate the response of MEFs to TNFα induced necrosis. Similar data were obtained by Remijsen et al. using a siRNA mediated knockdown of PGAM5 which did not block TNF induced necrosis in L929 cells. Similar results have been obtained when Drp1 was either silenced or knocked out (Moujalled et al., 2014; Remijsen et al., 2014). Again, in these experiments, Remijsen et al. utilized L929 cells. Moujalled et al. used DRP1^−/−^ MEFs to show that cells can undergo necroptosis independently of DRP1. We ourselves have also found that PGAM5 and Drp1 are not necessary for TNFα-induced necroptosis to occur in both MEFs and HT-29 cells (unpublished data). Perhaps the most elegant study indicating that mitochondria are entirely dispensable for necroptosis was a recent paper by Doug Green's group (Tait et al., 2013). Here they depleted SVEC and 3T3 cells of mitochondria via the induction of mitophagy by the uncoupler carbonylcyanide m-chlorophenylhydrazone (CCCP). They demonstrated that although TNFα-induced ROS was lost in the mitochondrially-deficient cells, necroptosis was still very much functional, whether it was induced by TNFα or more directly by RIP3 dimerization. However, this study was not without limitations since about 20% of cells studied still contained some mitochondria. Thus mitochondria were not completely eliminated and could still therefore have contributed, at least partially, to the necroptotic response. In future studies, it would be of value to sort cells that contain and do not contain mitochondria following induction of necroptosis to further confirm that this process is mitochondria-independent.

Consistent with these findings, very recent studies have emerged indicating that MLKL may bypass the mitochondria altogether, instead translocating to the plasma membrane upon homo-oligermerization (Cai et al., 2014; Chen et al., 2014). Cai et al. utilized HEK293 cells to initially show that MLKL oligomerizes upon induction of necrosis. This oligomerization was confirmed under more physiologically relevant conditions by inducing necroptosis in HT-29, Jurkat, U937, MEF, and J774A.1 cells, indicating that this process occurs in multiple cell types. To measure translocation to the plasma membrane, HEK293 and HT-29 cells were transfected with a fluorescent MLKL construct. These results were confirmed biochemically by isolating cell surface proteins from HT-29, MEF and J774A.1 cells. Chen et al. used HeLa cells to detect MLKL oligomerization and confirmed this result in L929 cells. MLKL translocation to the plasma membrane was measured in MLKL deficient L929 cells using a fluorescent MLKL construct and live cell imaging. It was proposed that plasmalemmal MLKL complexes then induced either Ca^2+^ (Cai et al., 2014) or Na^+^ (Chen et al., 2014) overload of the cell. However, new studies from Wang's and Vandenabeele's groups have demonstrated that MLKL can bind to phosphatidylinositides (PIPs) and can directly permeabilize liposomes containing these phospholipids (Dondelinger et al., 2014; Wang et al., 2014). Dondelinger et al. initially characterized MLKL's interaction with PIPs using a recombinant MLKL fragment and a lipid array and confirmed its pore forming abilities using liposomes loaded with MLKL. Finally, to confirm MLKL-PIP interactions in necroptosis, L929 and Jurkat cells were treated with PIP production inhibitors, which attenuated TNFα induced necrosis. It is important to note that the early cell free results of MLKL-PIP interactions were not confirmed in whole cells, but instead were modulated indirectly by pharmacologic intervention. Wang et al. confirmed MLKL translocation to the plasma membrane upon induction of necroptosis in HT-29 cells. MLKL-lipid interactions were tested using recombinant protein in a cell free system. And MLKL was shown to be able to form pores in liposomes. Again, however, these results were not confirmed in live cells. These data suggest that it is MLKL itself that is responsible for the final rupture of the plasma membrane during necroptosis and would again rule out a role for mitochondria.

## Conclusions

It is clear that our current understanding of programmed necrosis needs to change. These recent studies would indicate that the execution of programmed necrosis proceeds by a novel, yet to be characterized mitochondrially independent mechanism. However, inspection of all of the literature suggests that cell-specific contexts may occur where the mitochondrial and necroptotic pathways bisect to mediate necrosis. Moreover, even if these pathways are truly distinct, it is unlikely that they will function in isolation in disease. The question is then what is the relative contribution of each pathway to a given necrotic state and how do they coordinate at certain levels.

Additionally, the majority of necroptotic studies have been performed *in vitro* in a variety of cell types, and different cell types may execute necroptosis via different mechanisms. Multiple studies have shown that mitochondrial ROS is critical to the execution of necroptosis in L929 cells (He et al., 2009; Vanlangenakker et al., 2011; Ardestani et al., 2013), while HT29 and U937 cells seem to lack this requirement (Degterev et al., 2005; He et al., 2009). Disparate results have been obtained in MEFs with ROS being both implicated in Lin et al. (2004) and dispensable for Tait et al. (2013) necroptosis. It would be of value to repeat the experiments undertaken by Tait et al. in a cell line in which mitochondrial ROS has been implicated in necroptosis, such as L929 cells, to determine if there is a universal mechanism of necroptotic signaling or if the execution of this pathway is cell-type specific. Whether or not necroptosis requires ROS or mitochondria *in vivo* has yet to be fully determined. For that matter, a majority of the studies performed in the study of necrosome signaling have utilized immortalized cell lines, which begs the question, how well will these findings translate into primary culture systems? Additionally, Ca^2+^ overload induces necrotic cell death. Can this occur in a mitochondria free system? By answering these questions, the actual pathway of necroptosis can be determined. It is possible that there may be multiple routes to necroptosis, and that under certain conditions the mitochondria are not required for its execution.

### Conflict of interest statement

The authors declare that the research was conducted in the absence of any commercial or financial relationships that could be construed as a potential conflict of interest.

## Abbreviations

CYLD: cylindromatosis
CypD: cyclophilin-D
Drp1: dynamin-related protein 1
GLUD1: glutamate dehydrogenase 1
MLKL: mixed lineage kinase domain-like
MPT: mitochondrial permeability transition
RIP: receptor interacting protein
ROS: reactive oxygen species, TNFα, tumor necrosis factor-α, TNFR, tumor necrosis factor-α receptor
TRADD: TNFR1-associated death domain protein.
